# Sleep regularity is a stronger predictor of mortality risk than sleep duration: A prospective cohort study

**DOI:** 10.1093/sleep/zsad253

**Published:** 2023-09-21

**Authors:** Daniel P Windred, Angus C Burns, Jacqueline M Lane, Richa Saxena, Martin K Rutter, Sean W Cain, Andrew J K Phillips

**Affiliations:** Turner Institute for Brain and Mental Health, School of Psychological Sciences, Faculty of Medicine, Nursing and Health Sciences, Monash University, Melbourne, VIC, Australia; Turner Institute for Brain and Mental Health, School of Psychological Sciences, Faculty of Medicine, Nursing and Health Sciences, Monash University, Melbourne, VIC, Australia; Division of Sleep and Circadian Disorders, Brigham and Women’s Hospital, Boston, MA, USA; Program in Medical and Population Genetics, Broad Institute, Cambridge, MA, USA; Center for Genomic Medicine, Massachusetts General Hospital, Boston, MA, USA; Division of Sleep and Circadian Disorders, Brigham and Women’s Hospital, Boston, MA, USA; Program in Medical and Population Genetics, Broad Institute, Cambridge, MA, USA; Center for Genomic Medicine, Massachusetts General Hospital, Boston, MA, USA; Department of Anesthesia, Critical Care and Pain Medicine, Massachusetts General Hospital and Harvard Medical School, Boston, MA, USA; Division of Sleep and Circadian Disorders, Brigham and Women’s Hospital, Boston, MA, USA; Program in Medical and Population Genetics, Broad Institute, Cambridge, MA, USA; Center for Genomic Medicine, Massachusetts General Hospital, Boston, MA, USA; Department of Anesthesia, Critical Care and Pain Medicine, Massachusetts General Hospital and Harvard Medical School, Boston, MA, USA; Centre for Biological Timing, Division of Endocrinology, Diabetes and Gastroenterology, School of Medical Sciences, Faculty of Biology, Medicine and Health, Manchester Academic Health Science Centre, University of Manchester, Manchester, UK; Diabetes, Endocrinology and Metabolism Centre, NIHR Manchester Biomedical Research Centre, Manchester University NHS Foundation Trust, Manchester, UK; Turner Institute for Brain and Mental Health, School of Psychological Sciences, Faculty of Medicine, Nursing and Health Sciences, Monash University, Melbourne, VIC, Australia; Turner Institute for Brain and Mental Health, School of Psychological Sciences, Faculty of Medicine, Nursing and Health Sciences, Monash University, Melbourne, VIC, Australia

**Keywords:** actigraphy, circadian rhythms, cancer, cardiology, metabolic disease, mortality, cause of death, behavioral sleep medicine

## Abstract

Abnormally short and long sleep are associated with premature mortality, and achieving optimal sleep duration has been the focus of sleep health guidelines. Emerging research demonstrates that sleep regularity, the day-to-day consistency of sleep–wake timing, can be a stronger predictor for some health outcomes than sleep duration. The role of sleep regularity in mortality, however, has not been investigated in a large cohort with objective data. We therefore aimed to compare how sleep regularity and duration predicted risk for all-cause and cause-specific mortality. We calculated Sleep Regularity Index (SRI) scores from > 10 million hours of accelerometer data in 60 977 UK Biobank participants (62.8 ± 7.8 years, 55.0% female, median[IQR] SRI: 81.0[73.8–86.3]). Mortality was reported up to 7.8 years after accelerometer recording in 1859 participants (4.84 deaths per 1000 person-years, mean (±SD) follow-up of 6.30 ± 0.83 years). Higher sleep regularity was associated with a 20%–48% lower risk of all-cause mortality (*p* < .001 to *p* = 0.004), a 16%–39% lower risk of cancer mortality (*p* < 0.001 to *p* = 0.017), and a 22%–57% lower risk of cardiometabolic mortality (*p* < 0.001 to *p* = 0.048), across the top four SRI quintiles compared to the least regular quintile. Results were adjusted for age, sex, ethnicity, and sociodemographic, lifestyle, and health factors. Sleep regularity was a stronger predictor of all-cause mortality than sleep duration, by comparing equivalent mortality models, and by comparing nested SRI-mortality models with and without sleep duration (*p* = 0.14–0.20). These findings indicate that sleep regularity is an important predictor of mortality risk and is a stronger predictor than sleep duration. Sleep regularity may be a simple, effective target for improving general health and survival.

## Graphical Abstract

**Graphical Abstract:**
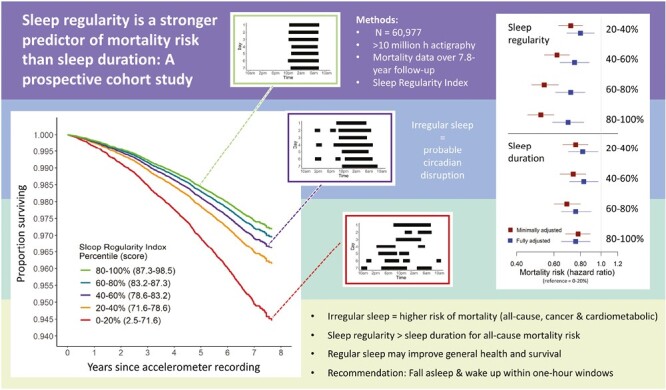

**Statement of Significance**

Sleep of adequate duration is important for optimal health and longevity, but emerging evidence demonstrates that regular sleep timing may be even more important. Using objective measures of sleep in a cohort of > 60 000 individuals, we found that people with less regular sleep patterns have a higher risk of premature mortality, and that sleep regularity is a stronger predictor of mortality risk than sleep duration. These findings were robust with detailed control for confounding factors, providing evidence that sleep regularity is a key index of human health and potentially a more important marker of health than sleep duration.

## Introduction

A large body of research has demonstrated that both subjective and objective estimates of average sleep duration are associated with mortality risk [1–9]. Several meta-analyses of subjective sleep duration have associated both short and/or long sleep duration, outside an approximate range of 7–9 hours, with higher risks for all-cause mortality [1–5]. Recently, large studies of objective sleep duration have confirmed these associations of sleep duration with all-cause mortality [6–9]. These findings are supported by results showing associations between sleep duration and many other dimensions of health [10].

Maintaining optimal sleep duration is the central focus of current sleep health guidelines [11]. Recent evidence, however, indicates that sleep regularity, defined as the day-to-day consistency of sleep–wake timing, is a stronger predictor of some health outcomes than average sleep duration [12, 13]. Studies with longitudinal measures of sleep regularity have found associations between irregular sleep and adverse cardiometabolic outcomes [12, 14–17], epigenetic aging [18], depressed mood [19], and lower quality of life [13]. People with irregular sleep patterns are exposed to irregular patterns of environmental stimuli, including light, and may have irregularly timed behaviors, such as physical activity and meals. This unstable timing of both stimuli and behaviors leads to disruption of circadian rhythms, with downstream negative health effects [20]. While mortality risk has been associated with self-reported sleep regularity [21], this relationship has not been investigated prospectively in a large cohort with objective sleep data. Furthermore, the relative importance of sleep duration compared with sleep regularity for mortality risk is not known.

We aimed to assess the relationship of objectively measured sleep regularity with risk for all-cause mortality, and mortality from cardiometabolic causes and cancer, in a large cohort (*N* = 60 977) who wore accelerometer devices for 1 week. We also assessed whether sleep regularity was a stronger predictor of mortality risk than sleep duration. We used data from our previously reported assessment of sleep regularity in UK Biobank participants [22], where we extracted Sleep Regularity Index (SRI) scores. This metric assesses day-to-day similarity in sleep patterns, accounting for irregularity due to fragmented sleep, napping, and variable sleep onset and offset timing.

## Methods

### Overview

A baseline cohort of approximately 502 000 participants aged between 40 and 69 years were recruited to the UK Biobank between 2006 and 2010 [23]. Participants completed an initial assessment at the time of recruitment, designed to capture information across a broad range of health and lifestyle factors, through questionnaires and physical measurements. Assessment centers were located to capture a range of socioeconomic, ethnic, and urban–rural distributions within the UK population. From this baseline cohort, 103 669 participants wore Axivity AX3 devices (Axivity, Newcastle upon Tyne, UK) on their dominant wrist for 7 days under free-living conditions between 2013 and 2016. Devices were tri-axial, and logged accelerometer data at 100 Hz. Invitations to participate and devices were distributed via post. Written informed consent was obtained and all data collection was conducted in accordance with the Declaration of Helsinki. See Supplementary Methods S1.1 for links to protocol and consent documents.

### Sleep regularity and sleep duration

Sleep regularity was assessed using the SRI [24], a metric that compares the similarity of sleep patterns from one day to the next. The SRI calculates the average concordance in sleep–wake state of all epoch pairs separated by 24 hours (see Supplementary Methods S1.7 for SRI calculation formula). An SRI of 100 represents perfectly regular sleep–wake patterns, and zero represents random patterns.

SRI scores were derived in this cohort in our previous work [22], and these scores were used in all analyses presented here. SRI scores were calculated using epoch-by-epoch sleep–wake state across each participant’s 1-week recording. Sleep–wake state was estimated using “GGIR”, a validated, widely used open-source R package for estimating sleep–wake from accelerometer data [25, 26], and “sleepreg”, an R package developed by our group to accurately calculate SRI scores from “GGIR” output [22]. The “sleepreg” package uses sustained inactivity data to account for naps, fragmented sleep, and large periods of wake during sleep in its calculation of SRI scores, which could not be achieved using “GGIR” summary output alone. This method accounted for device non-wear, and excluded instances of miscalculated sleep onset and offset. It also allowed patterns including more than one sleep episode within a 24-hour period to be accurately represented. Valid SRI scores were calculated in participants with at least 120 hours (i.e., 5 days) of 24-hour-separated epoch pairs after non-wear removal and exclusion of miscalculated days. Further detail is included in Supplementary Methods S1.6-8, and all scripts used in generating SRI scores are included in the “sleepreg” package, which is freely available on GitHub [https://github.com/dpwindred/sleepreg].

Sleep duration was calculated on a daily basis for each individual, as the duration of sustained inactivity between daily sleep onset and sleep offset times estimated by GGIR. Daily sleep durations were extracted in the same study days used to calculate SRI scores, and participant-level average sleep duration was calculated across these days. Similarly, participant-level average mid-sleep timing was calculated from daily mid-sleep, defined as the clock time halfway between sleep onset and sleep offset. Intraindividual variability in sleep onset and offset timing was calculated as the standard deviation of daily sleep onset and offset times for each individual.

### Mortality records

Mortality data were received from NHS Digital (England) and NHS Central Register (Scotland). Records include date of death and primary cause of death, diagnosed in accordance with the ICD-10 [27]. Records from June 2013 to March 2021 were included. Cardiometabolic mortality was defined according to ICD-10 diseases of the circulatory system, or endocrine and metabolic diseases (I05-I89, E00-E90). Predominant circulatory causes of death were ischemic heart disease (I20-I25); cerebrovascular diseases (I60-I69); other heart disease (I30-I52); diseases of the arteries, arterioles, and capillaries (I70-I79); and hypertensive diseases (I10-I15). Predominant endocrine and metabolic causes of death were diabetes mellitus (E10-E14); metabolic disorders (E70-E90); and obesity (E65-E68). Cancer was defined as any cause of death by malignant or benign neoplasms (C00-C97, D10-48). Predominant causes of death by cancer were malignant neoplasms of digestive organs (C15-C26); respiratory and intrathoracic organs (C30-C39); lymphoid and hematopoietic tissue (primary; C81-C96); and breast (C50).

### Covariates

Average physical activity was defined as average device acceleration across the recording, after exclusion of low-quality data and periods of non-wear, as described in previous work [28], and was derived from the same accelerometer records used to estimate sleep–wake state. Additional covariates were collected during an initial assessment between 2006 and 2010, including: self-reported ethnic background; employment status; yearly household income; Townsend Deprivation Index (average material deprivation of a participant’s residential location); weekly social activities; frequency of social visits; smoking status; urban or rural postcode; rotating shift work status; prescription of medication for hypertension or cholesterol; diagnosis of cancer, diabetes, or vascular conditions; body mass index (BMI); cholesterol ratio; frequency of depressed mood, unenthusiasm/disinterest, tenseness/restlessness, tiredness/lethargy; and visitation to a general practitioner or psychiatrist for mental health concerns. See Supplementary Methods S1.2-3 for detailed descriptions of covariates.

### Statistical analyses

SRI and sleep duration were split into quintiles. Hazards of mortality were estimated for each of the top four SRI and sleep duration quintiles compared to their respective lowest quintiles, which were hypothesized to have the highest mortality risk. This approach allowed for unspecified non-linearity in sleep/mortality relationships. Hazard ratios for all-cause and cause-specific mortality were estimated using Cox proportional hazards models and competing-risks proportional sub-hazards models [29]. All Cox models used time since accelerometer recording as the timescale. Sleep variables were included as predictors of mortality risk in three separate Cox models, as follows: model 1: SRI, model 2: sleep duration, and model 3: SRI plus sleep duration. Each of these three models were implemented as both “minimally adjusted” and “fully adjusted”, for a total of six models. Minimally adjusted models included age, sex, and ethnicity; and fully adjusted models were additionally adjusted for physical activity, employment, income, deprivation, social activities, social visits, smoking status, urbanicity, shift work status, and use of medication for cholesterol or hypertension (see Supplementary Methods S1.5 for model syntax). All covariates were selected as potential confounders of sleep/mortality relationships. Additional covariates in the fully adjusted models were also possible mediators of sleep/mortality relationships. Akaike Information Criteria was used to compare models 1 and 2 (equivalent SRI-only and sleep duration-only models). Likelihood ratio test was used to compare models 1 and 3 (nested hierarchical models). See Supplementary Results S2.2 for further detail on model comparisons.

Fully adjusted models 1–3 were subject to further adjustments for sleep timing, and baseline physical and mental health (see Supplementary Results S2.4-6). Each of the following variables were individually added to fully adjusted models 1–3: mid-sleep timing, cancer diagnosis, diabetes diagnosis, vascular conditions, BMI, cholesterol ratio, depressed mood, unenthusiasm/disinterest, tenseness/restlessness, tiredness/lethargy, and visitation to a GP or psychiatrist for mental health concerns.

The association between SRI and sleep duration was tested using linear regression, by inclusion of sleep duration as a linear, quadratic, and cubic predictor of SRI in three individual models. Model fit was compared between each of the three models using ANOVA (F-test). Non-linear relationships were tested due to the hypothesis that more irregular sleep would be associated with both shorter and longer sleep, primarily due to sleep restriction and associated recovery sleep.

## Results

### Participant characteristics

Our analyses included 60 997 participants with valid SRI scores. The SRI distribution was negatively skewed, had a median (IQR) of 81.0 (73.8–86.3), and ranged from 2.5 to 98.5, as described in our previous work [22]. Sleep duration had mean (±SD) of 6.77 ± 1.00 hours, and ranged from 0.48 to 13.49 hours. The mean (±SD) follow-up period between accelerometer recording and study endpoint (March 21, 2021) was 6.30 ± 0.83 years. All-cause mortality rate was 4.84 deaths per 1000 person-years, with 1859 all-cause deaths, 377 by cardiometabolic causes, and 1092 by cancer. Mean (±SD) time to mortality was 5.96 ± 1.32 years. Participants in the final sample of 60 997 were 62.8 ± 7.8 years of age, 55.0% female, 97.2% white ethnicity, 60.8% employed, 7.3% shift workers, and had median income range £31000–51 999, Townsend deprivation index score of −1.79 ± 2.77, ≥1 weekly social activities in 72.9%, social visits most commonly experienced weekly (36.2%), 83.6% from an urban postcode, 36.4% previous and 6.3% current smokers, and mean (±SD) physical activity of 28.4 ± 8.1 milli-g across weekly recordings. Descriptive statistics of each covariate across SRI and sleep duration quintiles are provided in Table 1. Number of deaths by all-causes, cardiometabolic, cancer, and other causes are provided in Tables 2 and 3 for each SRI and sleep duration quintile. Intraindividual variability in sleep onset and offset timing across SRI quintiles are provided in Supporting Information Supplementary Results S2.7.

**Table 1.** Descriptive statistics for participants, grouped according to sleep regularity and sleep duration percentiles

	Sleep regularity percentile		40%–60%	60%–80%	80%–100%	Sleep duration percentile		40%–60%	60%–80%	80%–100%
	0%–20%	20%–40%				0%–20%	20%–40%			
Age (years)										
M ± SD	63.14 ± 7.8	62.71 ± 7.83	62.34 ± 7.81	62.52 ± 7.72	63.17 ± 7.75	63.19 ± 7.84	62.24 ± 7.89	62.24 ± 7.78	62.79 ± 7.74	63.43 ± 7.61
Range	43.89–78.72	43.59–79.00	43.69–78.84	43.50–78.20	44.10–78.88	43.92–79.00	43.50–78.88	43.69–78.84	43.90–78.61	44.10–78.20
Sex (% male, *N*)	49.58 (6048)	46.23 (5640)	43.67 (5318)	42.68 (5203)	42.24 (5164)	57.51 (7015)	47.65 (5813)	42.75 (5215)	39.15 (4776)	37.33 (4554)
Ethnicity (% white, *N*)	95.39 (11 573)	96.55 (11 734)	97.27 (11 819)	97.92 (11 907)	98.76 (12 033)	94.58 (11 474)	96.89 (11 770)	97.55 (11 874)	98.07 (11 922)	98.81 (12 026)
Physical activity (milli-g)										
M ± SD	25.93 ± 7.97	27.68 ± 7.85	28.70 ± 7.80	29.42 ± 7.98	30.11 ± 8.08	28.65 ± 8.46	29.33 ± 8.23	28.93 ± 8.01	28.20 ± 7.75	26.74 ± 7.63
Range	4.83–67.81	6.48–69.41	6.46–69.21	7.91–68.61	8.05–68.64	5.69–68.64	7.34–69.41	4.83–67.23	6.46–68.64	5.52–69.21
Employment status (% employed, *N*)	58.33 (7052)	62.26 (7546)	62.98 (7625)	62.07 (7522)	58.45 (7108)	60.81 (7360)	63.8 (7735)	63.73 (7726)	59.93 (7266)	55.82 (6766)
Household income bracket (M ± SD, range 1–5)	2.65 ± 1.17	2.82 ± 1.16	2.91 ± 1.16	2.97 ± 1.14	2.94 ± 1.14	2.78 ± 1.17	2.92 ± 1.16	2.93 ± 1.15	2.87 ± 1.15	2.79 ± 1.14
*Townsend deprivation index*										
M ± SD	−1.33 ± 3.02	−1.68 ± 2.82	−1.84 ± 2.74	−1.96 ± 2.64	−2.14 ± 2.54	−1.4 ± 3.00	−1.69 ± 2.81	−1.87 ± 2.72	−1.94 ± 2.66	−2.06 ± 2.60
Range	−6.26–9.89	−6.26–8.94	−6.26–9.16	−6.26–10.46	−6.26–10.1	−6.26–10.46	−6.26–9.89	−6.26–9.89	−6.26–9.41	−6.26–10.1
Social visits (M ± SD, range 1–7)	5.15 ± 1.17	5.19 ± 1.12	5.18 ± 1.09	5.20 ± 1.08	5.14 ± 1.08	5.13 ± 1.16	5.16 ± 1.12	5.16 ± 1.10	5.20 ± 1.08	5.22 ± 1.08
Social activities (% >1 weekly, *N*)	70.29 (8553)	72.18 (8785)	73.68 (8965)	73.65 (8965)	74.56 (9105)	72.11 (8779)	74.21 (9040)	73.8 (8993)	73.05 (8892)	71.19 (8669)
Smoking										
% previous, *N*	39.10 (4751)	36.92 (4491)	36.29 (4412)	35.90 (4367)	33.84 (4127)	39.01 (4748)	36.46 (4433)	35.56 (4329)	35.71 (4343)	35.29 (4295)
% current, *N*	9.46 (1149)	6.90 (839)	5.95 (723)	4.77 (580)	4.22 (514)	8.59 (1045)	6.59 (801)	6.15 (749)	5.16 (628)	4.78 (582)
Urbanicity (% >10 000 population, *N*)	86.14 (9444)	83.88 (9317)	83.38 (9308)	82.85 (9288)	81.78 (9222)	85.23 (9409)	83.79 (9339)	83.51 (9337)	83.37 (9357)	82.09 (9137)
Shift work status (% shift workers, *N*)	10.37 (1252)	8.46 (1024)	7.00 (847)	5.80 (702)	5.12 (622)	9.55 (1154)	7.51 (910)	6.82 (826)	6.71 (813)	6.14 (744)
*SRI*										
M ± SD	62.03 ± 9.54	75.45 ± 1.97	80.95 ± 1.32	85.22 ± 1.2	90.20 ± 2.11	72.56 ± 12.32	78.85 ± 9.99	80.58 ± 9.49	81.25 ± 9.17	80.65 ± 9.69
Median (IQR)	65.10 (58.4–68.9)	75.62 (73.8–77.2)	80.99 (79.8–82.1)	85.22 (84.2–86.3)	89.80 (88.5–91.6)	74.59 (65.9–81.5)	80.66 (74.2–85.8)	82.45 (76.2–87.1)	82.98 (77.3–87.5)	82.63 (76.2–87.3)
Range	2.46–71.65	71.65–78.56	78.56–83.16	83.17–87.31	87.32–98.53	9.18–97.23	6.05–97.29	9.82–98.19	16.57–97.59	2.46–98.53
*Sleep duration (h)*										
M ± SD	6.25 ± 1.29	6.69 ± 1	6.86 ± 0.88	6.95 ± 0.81	7.07 ± 0.74	5.32 ± 0.72	6.33 ± 0.16	6.82 ± 0.13	7.29 ± 0.15	8.07 ± 0.47
Median (IQR)	6.32 (5.5–7.1)	6.70 (6.1–7.3)	6.86 (6.3–7.4)	6.95 (6.4–7.5)	7.07 (6.6–7.6)	5.54 (5.1–5.8)	6.34 (6.2–6.5)	6.82 (6.7–6.9)	7.28 (7.2–7.4)	7.94 (7.7–8.3)
Range	0.65–13.03	0.51–13.49	0.48–11.64	0.59–12.74	3.98–10.8	0.48–6.02	6.02–6.59	6.59–7.05	7.05–7.56	7.56–13.49

**Table 2.** Risk of all-cause, cardiometabolic, cancer and other-cause mortality, according to sleep regularity quintiles (model 1)

Model	Percentile	All-cause		Cardiometabolic		Cancer		Other-cause	
		*N* (%)	HR [95% CI]	*N* (%)	HR [95% CI]	*N* (%)	HR [95% CI]	*N* (%)	HR [95% CI]
Minimal	SRI (0%–20%)	565 (0.93)	—	130 (0.21)	—	294 (0.48)	—	140 (0.23)	—
*N* = 60 780	SRI (20%–40%)	389 (0.64)	0.72 [0.63 to 0.82]***	79 (0.13)	0.65 [0.49 to 0.86]**	230 (0.38)	0.81 [0.68 to 0.97]*	80 (0.13)	0.60 [0.46 to 0.79]***
	SRI (40%–60%)	322 (0.53)	0.62 [0.54 to 0.71]***	61 (0.1)	0.54 [0.40 to 0.73]***	204 (0.34)	0.75 [0.63 to 0.89]**	56 (0.09)	0.44 [0.33 to 0.61]***
	SRI (60%–80%)	284 (0.47)	0.54 [0.47 to 0.63]***	49 (0.08)	0.43 [0.31 to 0.60]***	182 (0.3)	0.66 [0.55 to 0.80]***	50 (0.08)	0.39 [0.29 to 0.55]***
	SRI (80%–100%)	288 (0.47)	0.52 [0.45 to 0.60]***	55 (0.09)	0.45 [0.33 to 0.61]***	176 (0.29)	0.61 [0.50 to 0.73]***	56 (0.09)	0.41 [0.30 to 0.56]***
Full	SRI (0%–20%)	434 (0.93)	—	96 (0.21)	—	231 (0.49)	—	106 (0.23)	—
*N* = 46 721	SRI (20%–40%)	296 (0.63)	0.80 [0.69 to 0.93]**	61 (0.13)	0.78 [0.56 to 1.08]	170 (0.36)	0.84 [0.69 to 1.02]	65 (0.14)	0.80 [0.58 to 1.09]
	SRI (40%–60%)	249 (0.53)	0.75 [0.64 to 0.88]***	50 (0.11)	0.72 [0.51 to 1.01]	155 (0.33)	0.83 [0.67 to 1.02]	44 (0.09)	0.63 [0.44 to 0.90]*
	SRI (60%–80%)	226 (0.48)	0.72 [0.61 to 0.84]***	45 (0.1)	0.69 [0.48 to 1.00]*	139 (0.3)	0.77 [0.62 to 0.95]*	39 (0.08)	0.60 [0.41 to 0.88]**
	SRI (80%–100%)	221 (0.47)	0.70 [0.59 to 0.83]***	41 (0.09)	0.62 [0.42 to 0.91]*	137 (0.29)	0.76 [0.61 to 0.94]*	42 (0.09)	0.66 [0.46 to 0.94]*

* *p* < .05, ** *p* < .01, *** *p* < .001. Minimal model was adjusted for age, sex, and ethnicity. Full model was adjusted for age, sex, ethnicity, physical activity, socioeconomic status, social visits, smoking status, urbanicity, shift work, use of medication for cholesterol or hypertension. Higher SRI quintiles represent more regular sleep.

**Table 3.** Risk of all-cause, cardiometabolic, cancer and other-cause mortality, according to sleep duration quintiles (model 2)

Model	Percentile	All-cause		Cardiometabolic		Cancer		Other-cause	
		*N* (%)	HR [95% CI]	*N* (%)	HR [95% CI]	*N* (%)	HR [95% CI]	*N* (%)	HR [95% CI]
Minimal	Duration (0%–20%)	502 (0.83)	—	129 (0.21)	—	257 (0.42)	—	114 (0.19)	—
*N* = 60 780	Duration (20%–40%)	340 (0.56)	0.76 [0.66 to 0.87]***	67 (0.11)	0.62 [0.46 to 0.83]**	200 (0.33)	0.86 [0.71 to 1.03]	72 (0.12)	0.72 [0.54 to 0.97]*
	Duration (40%–60%)	323 (0.53)	0.74 [0.64 to 0.85]***	64 (0.11)	0.62 [0.46 to 0.84]**	198 (0.33)	0.87 [0.72 to 1.04]	60 (0.1)	0.62 [0.45 to 0.85]**
	Duration (60%–80%)	314 (0.52)	0.69 [0.60 to 0.80]***	52 (0.09)	0.49 [0.35 to 0.67]***	204 (0.34)	0.86 [0.72 to 1.04]	58 (0.1)	0.57 [0.42 to 0.79]***
	Duration (80%–100%)	369 (0.61)	0.78 [0.68 to 0.89]***	62 (0.1)	0.55 [0.41 to 0.74]***	227 (0.37)	0.92 [0.77 to 1.10]	78 (0.13)	0.73 [0.55 to 0.98]*
Full	Duration (0%–20%)	380 (0.81)	—	98 (0.21)	—	196 (0.42)		85 (0.18)	—
*N* = 46 721	Duration (20%–40%)	260 (0.56)	0.82 [0.70 to 0.97]*	54 (0.12)	0.73 [0.52 to 1.02]	145 (0.31)	0.86 [0.69 to 1.06]	60 (0.13)	0.91 [0.66 to 1.27]
	Duration (40%–60%)	256 (0.55)	0.83 [0.71 to 0.97]*	48 (0.1)	0.67 [0.47 to 0.95]*	160 (0.34)	0.96 [0.78 to 1.19]	47 (0.1)	0.73 [0.51 to 1.04]
	Duration (60%–80%)	253 (0.54)	0.76 [0.65 to 0.90]**	45 (0.1)	0.59 [0.41 to 0.84]**	162 (0.35)	0.92 [0.74 to 1.13]	46 (0.1)	0.66 [0.46 to 0.95]
	Duration (80%–100%)	277 (0.59)	0.76 [0.65 to 0.89]***	48 (0.1)	0.56 [0.39 to 0.80]**	169 (0.36)	0.88 [0.72 to 1.09]	58 (0.12)	0.72 [0.51 to 1.02]

* *p* < .05, ** *p* < .01, *** *p* < .001. Minimal model was adjusted for age, sex, and ethnicity. Full model was adjusted for age, sex, ethnicity, physical activity, socioeconomic status, social visits, smoking status, urbanicity, shift work, use of medication for cholesterol or hypertension. Higher duration quintiles represent longer sleep duration.

### Sleep regularity is a stronger predictor of all-cause mortality than sleep duration

**Figure 1.**
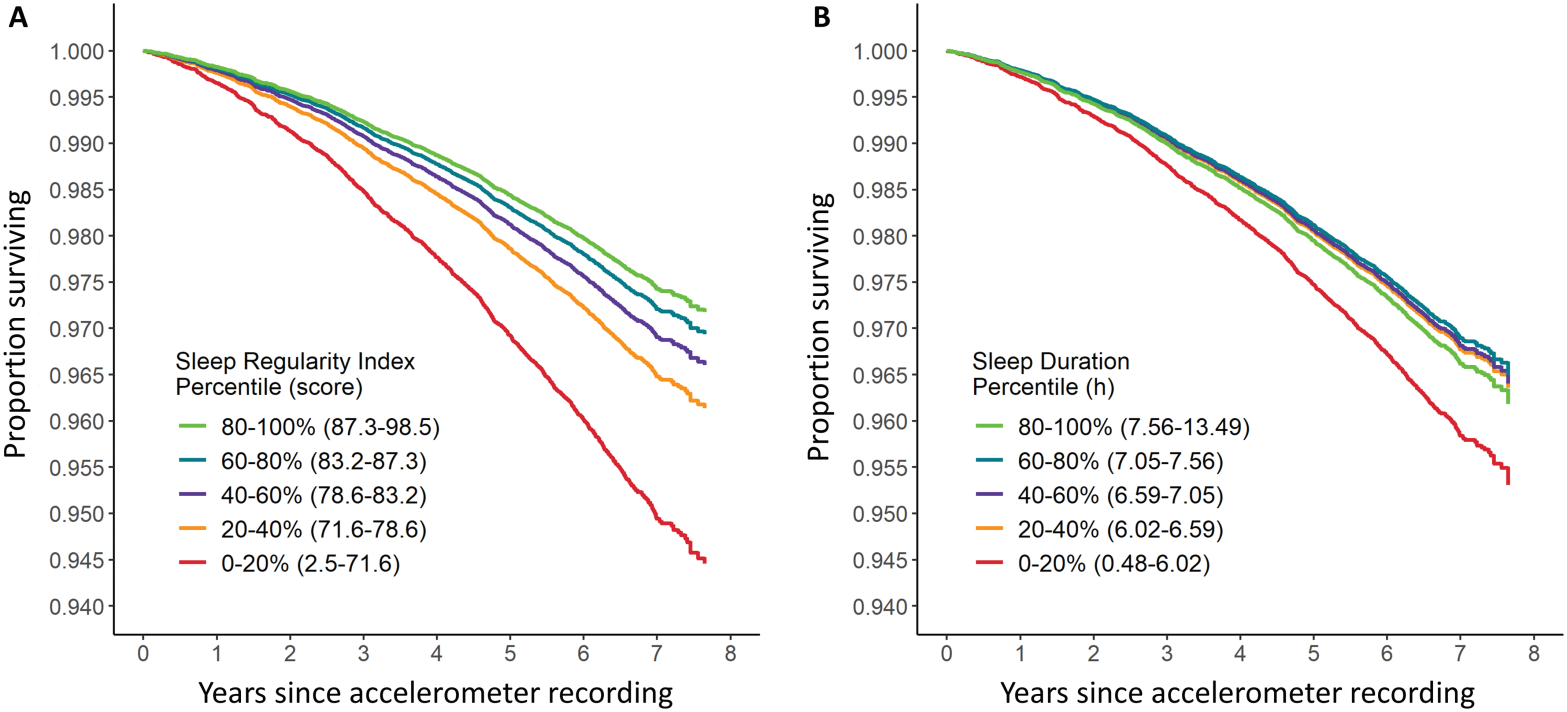
Cumulative survival of participants between accelerometer recording and study end point, according to (A) Sleep Regularity Index quintiles and (B) sleep duration quintiles.

Sleep regularity and mortality exhibited a monotonic relationship, with higher sleep regularity predicting lower risk of mortality in Cox proportional hazards models (Table 2, Figure 1, model 1). Individuals in the 80–100th sleep regularity percentiles had the lowest risk compared to the 0–20th percentiles (minimal model: HR = 0.52 [0.45–0.60], *p* < .001, full model: HR = 0.70 [0.59–0.83], *p* < .001).

Sleep duration showed a non-linear U-shaped relationship with all-cause mortality in the minimally adjusted Cox model, and a linear trend in relation to mortality in the fully adjusted model (Table 3, Figure 1, model 2). Longer sleep duration predicted lower risk of mortality up to the 60–80th percentiles in the minimal model (HR = 0.69 [0.60–0.80], *p* < .001), and up to the 80–100th percentiles in the fully adjusted model (HR = 0.76 [0.65–0.89], *p* < .001).

Minimum hazard ratios were lower in sleep regularity models (minimal: HR = 0.52 [0.45–0.60], *p* < .001; full: HR = 0.70 [0.59–0.83], *p* < .001) compared to sleep duration models (minimal: HR = 0.69 [0.60–0.80], *p* < .001, full: HR = 0.76 [0.65–0.89], *p* < .001), suggesting that sleep regularity is more strongly associated with mortality risk than sleep duration. Formal model comparisons (model 1 vs. model 2) revealed that sleep regularity models provided a better fit to all-cause mortality data than equivalent sleep duration models. The minimal and fully adjusted sleep duration models had small probabilities of minimizing information loss compared to corresponding sleep regularity models, calculated by comparing Akaike Information Criteria (minimal: *p* < .001; fully adjusted: *p* = .005; see Supplementary Results S2.2).

Sleep regularity remained a significant predictor of mortality risk in Cox models that also included sleep duration as a predictor (Table 4, model 3). The lowest risk of all-cause mortality was observed in the 80–100^th^ SRI percentiles in these SRI-duration models (minimally adjusted model: HR = 0.54 [0.47–0.63], *p* < .001, fully adjusted model: HR = 0.74 [0.62–0.89], *p* < .001), and the relationship between higher SRI and lower mortality risk remained monotonic. Hazard ratios for all SRI percentiles were slightly attenuated in SRI-duration models compared to SRI-only models presented in Table 2. In these SRI-duration models, sleep duration was a significant predictor of mortality for participants in the 60–80th percentiles (minimal: HR = 0.84 [0.73–0.98], *p* < .05; full: HR = 0.84 [0.71–0.99], *p* < .05) and 80–100th percentiles (full: HR = 0.84 [0.71–0.99], *p* < .05) compared to those in the 0–20th percentiles (Table 4). Hierarchical comparison of SRI-only (model 1) and SRI-duration (model 3) models indicated that the SRI-duration models were not a better fit for mortality risk compared to SRI-only models (likelihood ratio test, minimal models: Χ^2^(4) = 6.88, *p* = .14; fully adjusted models: Χ^2^(4) = 5.94, *p* = .20), suggesting that sleep duration does not explain significant additional variance in mortality risk beyond the variance explained by SRI scores.

**Table 4.** Risk of all-cause, cardiometabolic, cancer and other-cause mortality, for models that include both sleep regularity and sleep duration (model 3)

Model	Percentile	All-cause		Cardiometabolic		Cancer		Other-cause	
		*N* (%)	HR [95% CI]	*N* (%)	HR [95% CI]	*N* (%)	HR [95% CI]	*N* (%)	HR [95% CI]
Minimal	SRI (0%–20%)	565 (0.93)	—	130 (0.21)	—	294 (0.48)	—	140 (0.23)	—
*N* = 60 780	SRI (20%–40%)	389 (0.64)	0.73 [0.64 to 0.83]***	79 (0.13)	0.70 [0.52 to 0.92]*	230 (0.38)	0.81 [0.68 to 0.97]*	80 (0.13)	0.62 [0.47 to 0.82]***
	SRI (40%–60%)	322 (0.53)	0.64 [0.56 to 0.74]***	61 (0.1)	0.60 [0.44 to 0.82]**	204 (0.34)	0.75 [0.62 to 0.89]**	56 (0.09)	0.47 [0.34 to 0.64]***
	SRI (60%–80%)	284 (0.47)	0.56 [0.49 to 0.65]***	49 (0.08)	0.49 [0.35 to 0.69]***	182 (0.3)	0.66 [0.55 to 0.80]***	50 (0.08)	0.42 [0.30 to 0.58]***
	SRI (80%–100%)	288 (0.47)	0.54 [0.47 to 0.63]***	55 (0.09)	0.52 [0.38 to 0.73]***	176 (0.29)	0.60 [0.50 to 0.73]***	56 (0.09)	0.44 [0.32 to 0.60]***
	Duration (0%–20%)	502 (0.83)	—	129 (0.21)	—	257 (0.42)	—	114 (0.19)	—
	Duration (20%–40%)	340 (0.56)	0.87 [0.75 to 1.00]	67 (0.11)	0.73 [0.54 to 0.99]*	200 (0.33)	0.95 [0.79 to 1.14]	72 (0.12)	0.89 [0.66 to 1.20]
	Duration (40%–60%)	323 (0.53)	0.88 [0.77 to 1.02]	64 (0.11)	0.76 [0.55 to 1.04]	198 (0.33)	0.99 [0.82 to 1.20]	60 (0.1)	0.81 [0.59 to 1.11]
	Duration (60%–80%)	314 (0.52)	0.84 [0.73 to 0.98]*	52 (0.09)	0.61 [0.43 to 0.85]**	204 (0.34)	1.00 [0.83 to 1.21]	58 (0.1)	0.77 [0.55 to 1.06]
	Duration (80%–100%)	369 (0.61)	0.93 [0.81 to 1.08]	62 (0.1)	0.67 [0.49 to 0.92]*	227 (0.37)	1.06 [0.88 to 1.27]	78 (0.13)	0.95 [0.71 to 1.29]
Full	SRI (0%–20%)	434 (0.93)	—	96 (0.21)	—	231 (0.49)	—	106 (0.23)	—
*N* = 46 721	SRI (20%–40%)	296 (0.63)	0.83 [0.71 to 0.96]*	61 (0.13)	0.85 [0.61 to 1.17]	170 (0.36)	0.84 [0.69 to 1.03]	65 (0.14)	0.83 [0.61 to 1.13]
	SRI (40%–60%)	249 (0.53)	0.78 [0.66 to 0.92]**	50 (0.11)	0.81 [0.57 to 1.16]	155 (0.33)	0.83 [0.68 to 1.02]	44 (0.09)	0.67 [0.46 to 0.96]*
	SRI (60%–80%)	226 (0.48)	0.75 [0.64 to 0.89]**	45 (0.1)	0.80 [0.55 to 1.18]	139 (0.3)	0.77 [0.62 to 0.96]*	39 (0.08)	0.65 [0.44 to 0.95]*
	SRI (80%–100%)	221 (0.47)	0.74 [0.62 to 0.89]***	41 (0.09)	0.75 [0.50 to 1.13]	137 (0.29)	0.76 [0.61 to 0.95]*	42 (0.09)	0.73 [0.50 to 1.05]
	Duration (0%–20%)	380 (0.81)	—	98 (0.21)	—	196 (0.42)	—	85 (0.18)	—
	Duration (20%–40%)	260 (0.56)	0.88 [0.75 to 1.04]	54 (0.12)	0.77 [0.55 to 1.09]	145 (0.31)	0.91 [0.73 to 1.13]	60 (0.13)	1.00 [0.71 to 1.40]
	Duration (40%–60%)	256 (0.55)	0.90 [0.77 to 1.07]	48 (0.1)	0.72 [0.50 to 1.04]	160 (0.34)	1.04 [0.84 to 1.29]	47 (0.1)	0.82 [0.57 to 1.18]
	Duration (60%–80%)	253 (0.54)	0.84 [0.71 to 0.99]*	45 (0.1)	0.64 [0.44 to 0.94]*	162 (0.35)	1.00 [0.80 to 1.23]	46 (0.1)	0.75 [0.52 to 1.09]
	Duration (80%–100%)	277 (0.59)	0.84 [0.71 to 0.99]*	48 (0.1)	0.61 [0.42 to 0.89]**	169 (0.36)	0.96 [0.78 to 1.19]	58 (0.12)	0.82 [0.57 to 1.17]

* *p* < .05, ** *p* < .01, *** *p* < .001. Minimal model was adjusted for age, sex, and ethnicity. Full model was adjusted for age, sex, ethnicity, physical activity, socioeconomic status, social visits, smoking status, urbanicity, shift work, use of medication for cholesterol or hypertension. Higher SRI and duration quintiles represent more regular sleep and longer sleep duration, respectively.

Sleep regularity remained a significant predictor of all-cause mortality, and a stronger predictor than sleep duration, after additional adjustment for mid-sleep timing (see Supplementary Results S2.4). Sleep regularity also remained a significant predictor of all-cause mortality after additional adjustment for preexisting cancer, diabetes, vascular conditions (heart attack, stroke, angina, or hypertension), high BMI, high cholesterol ratio, depressed mood, unenthusiasm/disinterest, tenseness/restlessness, tiredness/lethargy, and visitation to a GP or psychiatrist for mental health concerns, in fully adjusted models, both with and without the inclusion of sleep duration (see Supplementary Results S2.5-6).

### Sleep regularity, sleep duration, and risk of cardiometabolic, cancer, and other-cause mortality

Sleep regularity was a significant predictor of cardiometabolic, cancer, and other-cause mortality risks in competing-risks proportional sub-hazards models (Table 2, model 1). The 60–80th and 80–100th sleep regularity percentiles had lower risk of cause-specific mortality compared to the 0–20th percentiles across all three causes, in both minimally and fully adjusted models. Largest effects for each outcome were: (1) cardiometabolic, minimal: HR = 0.43 [0.31–0.60], *p* < .001, full: HR = 0.62 [0.42–0.91], *p* < .05, (2) cancer, minimal: HR = 0.61 [0.50–0.73], *p* < .001, full: HR = 0.76 [0.61–0.94], *p* < .05, and (3) other-cause, minimal: HR = 0.39 [0.29–0.55], *p* < .001, full: HR = 0.60 [0.41–0.88], *p* < .01.

Sleep duration was a significant predictor of cardiometabolic mortality and other-cause mortality risks, but was not a significant predictor of cancer mortality risk (Table 3, model 2). The 40–60th, 60–80th, and 80–100th sleep duration percentiles had lower risks of cardiometabolic mortality compared to the 0–20th percentiles in both minimally and fully adjusted models. Only the 60–80th percentiles were significant predictors of other-cause mortality across both minimal and fully adjusted models. Largest effects for each outcome were: (1) cardiometabolic, minimal: HR = 0.49 [0.35–0.67], *p* < .001, full: HR = 0.56 [0.39–0.80], *p* < .01, and (2) other-cause, minimal: HR = 0.58 [0.42–0.79], *p* < .001, full: HR = 0.66 [0.46–0.95], *p* < .05.

Sleep regularity remained a significant predictor of cardiometabolic, cancer, and other-cause mortality risks after addition of sleep duration in the minimal model (Table 4, model 3). In the fully adjusted model, sleep regularity remained a significant predictor of cancer and other-cause mortality risk, but not cardiometabolic mortality, after addition of sleep duration.

### Sleep regularity associates with sleep duration

Sleep duration was a weak, significant predictor of SRI. Linear, quadratic, and cubic fits for this relationship were tested. The cubic model was a better fit than linear (F(2, 60 993) = 1360.9, *p* < .001) or quadratic (F(1, 60 993) = 426.5, *p* < .001), determined by ANOVA. In this model, longer sleep duration was associated with higher SRI scores up to a sleep duration of 7.83 hours (SRI of 81.76), above which longer sleep duration was associated with lower SRI. Adjusted R^2^ values for each model were 0.085 (linear), 0.118 (quadratic), and 0.125 (cubic). See Supplementary Results S2.3 for additional detail.

## Discussion

Across > 10 million hours of actigraphy data in 60 977 UK Biobank participants, we found that more regular sleep was a significant predictor of lower risk of all-cause mortality. Participants in the top four quintiles (SRI 71.6–98.5) had a 20%–48% lower risk of all-cause mortality compared to those with SRI scores in the bottom quintile (SRI < 71.6). Notably, sleep regularity was a stronger predictor of all-cause mortality than sleep duration. We assessed this by comparison of equivalent SRI-only and duration-only models, and by comparison of nested SRI-duration models. These results were robust to adjustment for age, sex, ethnicity, physical activity, smoking status, shift work status, sociodemographic and lifestyle factors, and additional adjustments for mid-sleep timing, preexisting cancer, diabetes, vascular events, high BMI, high cholesterol ratio, and baseline mental health.

We developed the SRI metric specifically to assess circadian disruption by measuring changes that occur to sleep patterns on a circadian timescale [24]. The SRI captures irregularity due to a combination of fragmented sleep, variable onset, offset, duration, and daytime napping [30]. In this respect, it is distinguished from other markers of intraindividual variability in sleep, which tend to assess only one dimension (e.g. variability in sleep onset timing), make assumptions about sleep patterns (e.g. only one sleep episode per day, as in the “composite phase deviation” metric), and assess intraindividual variability across the entire study period, rather than on a day-to-day basis (e.g. “inter-daily stability”) [30]. Consequently, the SRI is likely to be both a sensitive measure of circadian disruption, contributing to poor long-term health outcomes, and a potential marker of the future development of poor health outcomes via the effects of irregularity on sleep.

Recent research has shown that the SRI is predictive of a wide range of health outcomes [12–19]. Our study establishes a relationship between SRI and mortality risk using objective data from accelerometers. This result aligns with previous accelerometer studies in smaller samples that have linked variability in specific dimensions of sleep with higher mortality risk, including sleep duration [31], and sleep fragmentation and awakenings [32, 33]. Our results also align with the previous finding that lower relative amplitude, a measure of the robustness of rest-activity rhythms, is associated with higher mortality risk [34, 35]. Furthermore, self-report studies have linked higher mortality risk with irregular sleep patterns [21], daytime napping [36, 37], and longitudinal variability in sleep duration [6]. Our results show definitively in a large prospective sample that irregular sleep is predictive of greater risk of mortality.

Many studies have linked both short sleep duration and long sleep duration with mortality risk [1–9]. Our results for short sleepers are consistent with this literature. We note that, in our analysis, the longest sleeping group corresponded to a sleep duration of > 7.56 hours, whereas previous studies showing an association of mortality with long sleep have tended to use more extreme cutoffs (e.g. >9 hours or the highest possible response self-report category). We therefore would not necessarily expect to see a heightened risk of mortality in this upper quintile.

Our study advances previous literature by directly comparing sleep duration and sleep regularity as predictors of mortality risk. Our findings demonstrate that sleep regularity is generally a stronger predictor of mortality risk than sleep duration. These findings align with other studies showing sleep regularity to be a stronger predictor than sleep duration of other health outcomes [12, 13]. We propose that these findings emerge because sleep regularity is a more direct proxy for circadian disruption, which is known from experimental studies to have very broad adverse effects on physiology [38]. In animal studies, circadian disruption induced by light patterns that contribute to irregular sleep–wake behavior is known to cause premature mortality [39] and purposeful disruption of the circadian clock with light causes profound cardiovascular disease [40]. Prospective studies of people with irregular sleep have demonstrated links with cardiometabolic health [12, 14–17]. We note that while sleep regularity is a more direct measure of circadian disruption, sleep duration may in part also be capturing aspects of circadian disruption. Short and long sleep duration may influence the timing of light exposure, nutritional intake, and physical activity, which impact both central and peripheral circadian rhythms.

Higher risk of mortality by cardiometabolic causes was associated with both irregular sleep and short sleep duration in our study. These findings are consistent with experimental and epidemiological evidence linking sleep regularity and duration with cardiometabolic health [4, 8, 12, 21, 41–46]. Experimental evidence in humans indicates that cardiometabolic risk factors, including arterial blood pressure, inflammatory markers, and insulin sensitivity are altered with reduced sleep duration [41], and under experimental conditions that disrupt circadian rhythms [42]. Epidemiological studies show that the risks of cardiovascular events and mortality are higher in short sleepers [4, 8, 43, 44] across both objective and subjective measures of sleep duration. For sleep regularity, epidemiological studies using actigraphy have linked more variable sleep timing and duration with incident fatal and non-fatal cardiovascular disease [45] and metabolic abnormalities [46], and SRI-measured sleep regularity with cardiometabolic risk factors [12]. Subjective sleep regularity has also been linked with cardiovascular mortality [21]. While sleep duration and regularity independently predicted cardiometabolic mortality risk in our study, we also found that regularity was not a significant predictor of cardiometabolic mortality in models that also included duration, after adjustment for physical activity, smoking status, shift work status, and sociodemographic and lifestyle factors. This result suggests that different aspects of sleep behaviors (duration vs. regularity) may differentially contribute to cardiometabolic mortality risk, possibly due to different effects of homeostatic and circadian disruption on cardiometabolic factors [41, 42].

We found that irregular sleep predicted higher risk of mortality by cancer, whereas short sleep duration did not. Similar to all-cause mortality, this relationship may be ultimately driven by circadian disruption. Several lines of evidence have linked circadian disruption with cancer, including: (1) studies implicating gene expression and metabolic rhythms in cancer initiation and progression [47]; (2) animal studies showing irregular sleep–wake rhythms and circadian disruption cause increased tumor progression and metastasis [48–51] and cancer-induced inflammation [52]; and (3) epidemiological evidence implicating light exposure at night in higher risk of incident breast [53–55], thyroid [56], and pancreatic cancer [57], and shift work exposure in breast [58], prostate [59], lung, and skin cancer [60]. In contrast, epidemiological evidence indicates that sleep duration is only linked with higher cancer mortality risk in a small substrata of long sleepers (>9 hours), but not in short sleepers [1, 43, 61], which is consistent with our findings for short sleepers. Notably, the relationship we found between irregular sleep and higher cancer mortality risk remained robust in individuals without a preexisting cancer diagnosis, further supporting the hypothesis that irregular sleep and circadian disruption influence cancer initiation, progression, and eventual mortality.

There are several limitations in this study. First, the single week of data collected for each individual provides only a snapshot of their sleep–wake patterns, and future work should collect sleep–wake data over a longer timeframe and include multiple weekend-weekday transitions. It is nevertheless interesting that even a snapshot of sleep behaviors is predictive of mortality for a follow-up period of several years. Second, accelerometer recordings did not occur simultaneously with collection of baseline covariates, and some of these covariates may not remain temporally stable within each individual. Third, our findings are within an older age group of mostly homogeneous ethnicity, and should be replicated across other cohorts, including cross-culturally. Fourth, our fully adjusted models contain variables that potentially have both confounding and mediating effects (e.g. smoking status). The true strength of the sleep regularity-mortality relationship therefore likely lies between our minimally adjusted models, which contain only non-mediating covariates, and our fully adjusted models. Finally, we acknowledge the correlational nature of our findings. Sleep regularity may be both a cause and marker of premature mortality risk.

One key determinant of a high SRI score is regular timing of sleep onset and offset. In our study, people with the top 20% of SRI scores went to sleep and awoke within approximately 1-hour windows on the majority of their study days. By contrast, people with the bottom 20% of SRI scores went to sleep and awoke within approximately 3-hour windows. Aiming to fall asleep and wake up within 1-hour windows each day may therefore be a feasible strategy for improving SRI, especially for individuals who keep only one main sleep episode per day. We note that awakenings during the night and fragmented sleep patterns also contribute to low SRI scores, and should therefore also be taken into consideration.

In summary, our findings challenge the long-standing assumption that sleep duration is the most important index of sleep for human health. The preponderance of research on sleep and health has focused on sleep duration as the primary predictor. The resultant frameworks for public health have consequently tended to focus on sleep duration as the key target. Our results confirm an important role for sleep duration in predicting mortality, but reveal that sleep regularity is an even stronger predictor. Fortunately, sleep regularity may also be an easier dimension to target through interventions. Due to both psychosocial and biological reasons, extending sleep duration can be challenging to achieve in practice. Interventions to extend sleep that do not directly schedule when participants can sleep have only small effects on sleep duration [62]. Asking people to maintain more similar sleep times between days to improve sleep regularity, rather than devoting a greater proportion of the day to sleep, may ultimately be a more feasible strategy.

## Supplementary Material

zsad253_suppl_Supplementary_Material

## Data Availability

The data underlying this article are available in the UK Biobank repository and can be accessed upon application (https://www.ukbiobank.ac.uk/).
