# Supplementary material for: Sleep regularity is a stronger predictor of mortality risk than sleep duration: A prospective cohort study
Source: Sleep. 2023 Sep 21;47(1):zsad253. doi: 10.1093/sleep/zsad253 (PMC10782501; doi:10.1093/sleep/zsad253)
Supplement: zsad253_suppl_Supplementary_Material [file zsad253_suppl_supplementary_material.docx]

**Supporting Information**

**Sleep regularity is a stronger predictor of mortality risk than sleep duration: A prospective cohort study**

Daniel P. Windred^1^, Angus C. Burns^1,2,4^, Jacqueline M. Lane^2-4^, Richa Saxena^2-4^, Martin K. Rutter^5,6^, Sean W. Cain^1^*, & Andrew J. K. Phillips^1^*

Affiliation / Institution:

^1^Turner Institute for Brain and Mental Health, School of Psychological Sciences, Faculty of Medicine, Nursing and Health Sciences, Monash University, Melbourne, VIC, Australia

^2^Center for Genomic Medicine, Massachusetts General Hospital, Boston, MA, USA

^3^Department of Anesthesia, Critical Care and Pain Medicine, Massachusetts General Hospital and Harvard Medical School, Boston, MA, USA

^4^Division of Sleep and Circadian Disorders, Brigham and Women’s Hospital, Boston, MA, USA

^5^Centre for Biological Timing, Division of Endocrinology, Diabetes & Gastroenterology, School of Medical Sciences, Faculty of Biology, Medicine and Health, Manchester Academic Health Science Centre, University of Manchester, Manchester, UK

^6^Diabetes, Endocrinology and Metabolism Centre, NIHR Manchester Biomedical Research Centre, Manchester University NHS Foundation Trust, Manchester, UK.

*Authors contributed equally to this manuscript.

Corresponding Authors: Daniel P. Windred, Andrew J. K. Phillips

Address: 264 Ferntree Gully Rd, Notting Hill, VIC 3168, Australia

[daniel.windred@monash.edu](mailto:daniel.windred@monash.edu)

andrew.phillips@monash.edu

**Contents**

[**S1. Supplementary Methods** 3](#_Toc141457942)

[**Table S1.1.** UK Biobank protocol documents 3](#_Toc141457943)

[**Table S1.2.** UK Biobank variables 4](#_Toc141457944)

[**Table S1.3.** Covariates derived from UK Biobank variables 8](#_Toc141457945)

[**Methods S1.4.** Cox Proportional Hazards and Proportional Sub-hazards models 10](#_Toc141457946)

[**Table S1.5.** Syntax for Models 1-3, minimal and fully-adjusted 11](#_Toc141457947)

[**S1.6.** Sleep-wake state estimation 12](#_Toc141457948)

[**S1.7.** Sleep Regularity Index (SRI) calculation 13](#_Toc141457949)

[**S1.8.** Cleaning and exclusions 14](#_Toc141457950)

[**S2. Supplementary Results** 15](#_Toc141457951)

[**Figure S2.1.** Cumulative survival of participants (fully-adjusted) 15](#_Toc141457952)

[**Results S2.2.** Comparison of equivalent sleep regularity and sleep duration models 16](#_Toc141457953)

[**Table S2.3.** Association between SRI and sleep duration 17](#_Toc141457954)

[**Table S2.5.** Hazard of mortality after adjustment for baseline physical health 19](#_Toc141457955)

[**Table S2.6.** Hazard of mortality after adjustment for baseline mental health 20](#_Toc141457956)

[**Table S2.7.** Intra-individual variability in sleep onset and offset timing according to SRI percentile 21](#_Toc141457957)

[**S3.** STROBE Checklist 22](#_Toc141457958)

[**References** 26](#_Toc141457959)

# **S1. Supplementary Methods**

| **Table S1.1.** UK Biobank protocol documents | |
| --- | --- |
| **Document** | **Link** |
| Invitation to participate | <https://biobank.ctsu.ox.ac.uk/crystal/refer.cgi?id=100253> |
| Accelerometer: Participant instructions | <https://biobank.ndph.ox.ac.uk/showcase/refer.cgi?id=141141> |
| Accelerometer: Collection and processing | <https://biobank.ndph.ox.ac.uk/showcase/refer.cgi?id=131600> |
| Death registry linkage | <https://biobank.ctsu.ox.ac.uk/crystal/refer.cgi?id=115559> |
| Assessment centre: Consent | <https://biobank.ndph.ox.ac.uk/showcase/refer.cgi?id=100230> |
| Assessment centre: Reception | <https://biobank.ndph.ox.ac.uk/showcase/ukb/docs/Reception.pdf> |

| **Table S1.2.** UK Biobank variables | | | | |
| --- | --- | --- | --- | --- |
| **Variable** | **Collection method** | **UKB ID** | **Description** | **Link** |
| Age | Registry | 21003 | Participant age at assessment centre visit (years). Obtained from NHS Primary Care Trust registries, and amended by participants at assessment centre visit if required. | <https://biobank.ndph.ox.ac.uk/showcase/field.cgi?id=21003> |
| Sex | Registry | 31 | Obtained from NHS Primary Care Trust registries, and amended by participants at assessment centre visit if required. | <https://biobank.ndph.ox.ac.uk/showcase/field.cgi?id=31> |
| Ethnic background | Assessment centre visit: Questionnaire | 21000 | Ethnic group (white, mixed, Asian/Asian British, black/black British, Chinese, other, prefer not to answer) and ethnic background (sub-categories within each ethnic group). | <https://biobank.ndph.ox.ac.uk/showcase/field.cgi?id=21000> |
| Physical activity | Accelerometer | 90012 | Average acceleration of Axivity AX3 device across one week collection | <https://biobank.ndph.ox.ac.uk/showcase/field.cgi?id=90012> |
| Current employment status | Assessment centre visit: Questionnaire | 6142 | Paid employment, retired, home/family caretaker, unable to work, unemployed, volunteer, student, other | <https://biobank.ndph.ox.ac.uk/showcase/field.cgi?id=6142> |
| Average total household income before tax | Assessment centre visit: Questionnaire | 738 | Income brackets: <£18k, £18k-£29.9k, £30k-£51.9k, £52k-£100k, >£100k, do not know, prefer not to answer | <https://biobank.ndph.ox.ac.uk/showcase/field.cgi?id=738> |
| Townsend deprivation index | Registry | 189 | Scores corresponding to average home ownership, car ownership, household overcrowding, and employment rate of each participant’s postcode. Calculated based on national census data at time of recruitment. | <https://biobank.ndph.ox.ac.uk/showcase/field.cgi?id=189> |
| Leisure / social activities | Assessment centre visit: Questionnaire | 6160 | One or more weekly social activities: Sports club /gym, pub or social club, adult education, other group activity, none, prefer not to answer | <https://biobank.ndph.ox.ac.uk/showcase/field.cgi?id=6160> |
| Frequency of friend/family visits | Assessment centre visit: Questionnaire | 1031 | Frequency of visits from family/friends: Almost daily, 2-4times a week, once a week, once a month, once every few months, never/almost never, no friends/family outside household, do not know, prefer not to answer | <https://biobank.ndph.ox.ac.uk/showcase/field.cgi?id=1031> |
| Smoking status | Assessment centre visit: Questionnaire | 20116 | Never, previous, current, prefer not to answer | <https://biobank.ndph.ox.ac.uk/showcase/field.cgi?id=20116> |
| Urbanicity | Registry | 20118 | ‘Urban’ (population ≥ 10,000) and ‘non-urban’ (population < 10,000), defined according to postcode population density records from the UK Office for National Statistics. | <https://biobank.ndph.ox.ac.uk/showcase/field.cgi?id=20118> |
| Shift work | Assessment centre visit: Questionnaire | 826 | Never, sometimes, usually, always, do not know, prefer not to answer. Answers in response to 'Does your work involve shift work?' | <https://biobank.ndph.ox.ac.uk/showcase/field.cgi?id=826> |
| Medication | Assessment centre visit: Questionnaire | 6153, 6177 | Cholesterol lowering, blood pressure lowering, insulin, hormone replacement therapy, oral contraceptive pill, none, do not know, prefer not to answer. Answers in response to ‘Do you regularly take any of the following medications?’ | <https://biobank.ndph.ox.ac.uk/showcase/refer.cgi?id=100572>  <https://biobank.ndph.ox.ac.uk/showcase/field.cgi?id=6177> |
| Diabetes diagnosed by a doctor | Assessment centre visit: Questionnaire | 2443 | Yes, no, do not know, prefer no to answer. Answers in response to 'Has a doctor ever told you that you have diabetes?' | <https://biobank.ndph.ox.ac.uk/showcase/field.cgi?id=2443> |
| Vascular condition diagnosed by a doctor | Assessment centre visit: Questionnaire | 6150 | Heart attack, angina, stroke, high blood pressure, none, prefer not to answer. Answers in response to ‘Has a doctor ever told you that you have had any of the following conditions?’ | <https://biobank.ndph.ox.ac.uk/showcase/field.cgi?id=6150> |
| Cancer diagnosed by a doctor | Assessment centre visit: Questionnaire | 2453 | Yes, no, do not know, prefer no to answer. Answers in response to 'Has a doctor ever told you that you have had cancer?' | <https://biobank.ndph.ox.ac.uk/showcase/field.cgi?id=2453> |
| Body mass index (BMI) | Assessment centre visit: Physical | 21001 | BMI = weight / (standing height)^2^ | <https://biobank.ndph.ox.ac.uk/showcase/field.cgi?id=21001> |
| Triglycerides | Assessment centre visit: Physical | 30870 | Blood biochemistry: concentration of triglycerides (mmol/L) | <https://biobank.ndph.ox.ac.uk/showcase/field.cgi?id=30870> |
| Low-density lipoprotein | Assessment centre visit: Physical | 30780 | Blood biochemistry: concentration of low-density lipoprotein (mmol/L) | <https://biobank.ndph.ox.ac.uk/showcase/field.cgi?id=30780> |
| High-density lipoprotein | Assessment centre visit: Physical | 30760 | Blood biochemistry: concentration of high-density lipoprotein (mmol/L) | <https://biobank.ndph.ox.ac.uk/showcase/field.cgi?id=30760> |
| Frequency of depressed mood | Assessment centre visit: Questionnaire | 2050 | Not at all, several days, more than half the days, nearly every day, do not know, prefer not to answer. Answers in response to ‘Over the past two weeks, how often have you felt down, depressed, and hopeless?’ | <https://biobank.ndph.ox.ac.uk/showcase/field.cgi?id=2050> |
| Frequency of unenthusiasm / disinterest | Assessment centre visit: Questionnaire | 2060 | Not at all, several days, more than half the days, nearly every day, do not know, prefer not to answer. Answers in response to ‘Over the past two weeks, how often have you had little interest or pleasure in doing things?’ | <https://biobank.ndph.ox.ac.uk/showcase/field.cgi?id=2060> |
| Frequency of tenseness / restlessness | Assessment centre visit: Questionnaire | 2070 | Not at all, several days, more than half the days, nearly every day, do not know, prefer not to answer. Answers in response to ‘Over the past two weeks, how often have you felt tense, fidgety, or restless?’ | <https://biobank.ndph.ox.ac.uk/showcase/field.cgi?id=2070> |
| Frequency of tiredness / lethargy | Assessment centre visit: Questionnaire | 2080 | Not at all, several days, more than half the days, nearly every day, do not know, prefer not to answer. Answers in response to ‘Over the past two weeks, how often have you felt tired or had little energy?’ | <https://biobank.ndph.ox.ac.uk/showcase/field.cgi?id=2080> |
| Visited GP for mental health | Assessment centre visit: Questionnaire | 2090 | Yes, no, do not know, prefer no to answer. Answers in response to 'Have you ever seen a general practitioner (GP) for nerves, anxiety, tension, or depression?’ | <https://biobank.ndph.ox.ac.uk/showcase/field.cgi?id=2090> |
| Visited psychiatrist for mental health | Assessment centre visit: Questionnaire | 2100 | Yes, no, do not know, prefer no to answer. Answers in response to 'Have you ever seen a psychiatrist for nerves, anxiety, tension, or depression?’ | <https://biobank.ndph.ox.ac.uk/showcase/field.cgi?id=2100> |

| **Table S1.3.** Covariates derived from UK Biobank variables | | |
| --- | --- | --- |
| **Covariate** | **UKB ID(s)** | **Description** |
| Age | 21003 | Continuous, age at accelerometer recording (years) |
| Sex | 31 | Binary, male = 1, female = 0 |
| Ethnicity | 21000 | Binary, white ethnic group = 1, other ethnic group = 0 |
| Employment status | 6142 | Binary, employed = 1, other categories = 0 |
| Income | 738 | Ordered categories, <£18k = 1, £18k-£29.9k = 2, £30k-£51.9k = 3, £52k-£100k = 4, and >£100k = 5. |
| Deprivation | 189 | Continuous, included as recorded |
| Social activities | 6160 | Binary, >0 weekly activities = 1, 0 weekly activities = 0 |
| Social visits | 1031 | Ordered categories: ‘no friends / family’ = 1 ‘never or almost never’ = 2, ‘every few months’ = 3, ‘monthly’ = 4, ‘weekly’ = 5, ‘2-4 times a week’ = 6, and ‘almost daily’ = 7 |
| Smoking status | 20116 | Categorical, 'never' (referent category), ‘previous’, and ‘current’ |
| Urbanicity | 20118 | Binary, urban = 1, rural = 0 |
| Shift work | 826 | Binary, 'Always' = 1, all other categories = 0 |
| Diabetes diagnosis | 2443 | Binary, 'Yes' = 1, 'No' = 0 |
| Vascular diagnosis | 6150 | Binary, ‘Heart attack’, and/or ‘Stroke’, and/or ‘Angina’ and/or ‘Hypertension’ = 1, 'None' = 0 |
| Cancer diagnosis | 2453 | Binary, 'Yes' = 1, 'No' = 0 |
| Physical activity | 90012 | Continuous, included as recorded |
| Medication | 6153, 6177 | Categorical, ‘no medication’ (referent), ‘hypertension medication’, ‘cholesterol medication’, and ‘hypertension & cholesterol medication’ |
| BMI | 21001 | Binary, high = 1, low = 0. ‘High’ defined as BMI > 30. |
| Cholesterol ratio | 30870, 30780, 30760 | Binary, high = 1, low = 0. Cholesterol ratio = (LDL + HDL + 0.2*(triglycerides))/HDL. ‘High’ defined as cholesterol ratio > 3.75 in males, and cholesterol ratio > 3.00 in females. |
| Depressed mood | 2050 | Binary, any depressed mood = 1, no depressed mood = 0. |
| Unenthusiasm / disinterest | 2060 | Binary, any unenthusiasm / disinterest = 1, no unenthusiasm / disinterest = 0. |
| Tenseness / restlessness | 2070 | Binary, any tenseness / restlessness = 1, no tenseness / restlessness = 0. |
| Tiredness / lethargy | 2080 | Binary, any tiredness / lethargy = 1, no tiredness / lethargy = 0. |
| Visited GP for mental health | 2090 | Binary, 'Yes' = 1, 'No' = 0 |
| Visited psychiatrist for mental health | 2100 | Binary, 'Yes' = 1, 'No' = 0 |

## **Methods S1.4. Cox Proportional Hazards and Proportional Sub-hazards models**

Cox proportional hazards models were implemented with the ‘survival’ package in R (version 4.1.0). Competing risks models for proportional sub-hazards were implemented with the ‘cmprsk’ package. Plotted survival curves were adjusted using the ‘survminer’ package using a ‘marginal’ approach, which balances each subgroup across all covariates. Contrasts for all categorical variables were set using ‘contr.treatment’, which defines a referent category to which all other categories are compared (e.g., 0-20^th^ percentile SRI score was defined as referent category, and 20-40^th^, 40-60^th^, 60-80^th^, and 80-100^th^ were comparison categories).

Syntax for the six models used in the primary analyses are presented in Table S1.5. These models tested the relationships of sleep regularity and sleep duration with all-cause mortality. Equivalent versions of the six models were also implemented for each cause-specific analysis (cancer, cardiometabolic, and other cause mortality), using a proportional sub-hazards modelling approach for competing risks.

Syntax for proportional sub-hazards models was as follows:

*crr(ftime = time, fstatus = death_cause, cov1 = covs, failcode = n)*

In this model, *time* represented the time between accelerometer recording and either death or censoring, *death_cause* was a numerical representation of cause of death (0 = living, 1 = cancer, 2 = cardiometabolic, 3 = other cause), *covs* was data structure that contained covariates (e.g., age, sex, ethnicity), and *n* represented the cause of death defined as the competing hazard in each model (e.g., when n = 1, competing hazard of mortality by cancer was tested).

The assumption of no multi-collinearity of model covariates was assessed by calculating the Variance Inflation Factor (VIF) using the ‘ols_vif_tol’ function. The assumption of proportional hazards was assessed by plotting the temporal stability of regression coefficients for each covariate, using the ‘cox.zph’ function.

| **Table S1.5.** Syntax for Models 1-3, minimal and fully-adjusted | | |
| --- | --- | --- |
| **Minimally adjusted** | SRI (Model 1) | *Surv(time, death) ~ SRI + age + sex + ethnicity* |
|  | Duration (Model 2) | *Surv(time, death) ~ sleep_duration + age + sex + ethnicity* |
|  | SRI + Duration (Model 3) | *Surv(time, death) ~ SRI + sleep_duration + age + sex + ethnicity* |
| **Fully adjusted** | SRI (Model 1) | *Surv(time, death) ~ SRI + age + sex + ethnicity + physical_activity + employment + income + deprivation + social_activity + social_visits + smoking + urbanicity + shiftwork + medication* |
|  | Duration (Model 2) | *Surv(time, death) ~ sleep_duration + age + sex + ethnicity + physical_activity + employment + income + deprivation + social_activity + social_visits + smoking + urbanicity + shiftwork + medication* |
|  | SRI + Duration (Model 3) | *Surv(time, death) ~ SRI + sleep_duration + age + sex + ethnicity + physical_activity + employment + income + deprivation + social_activity + social_visits + smoking + urbanicity + shiftwork + medication* |
| In each model, *time* represents the time between accelerometer recording and either death or censoring, and *death* is a binary marker of participant survival. | | |

## **S1.6. Sleep-wake state estimation**

Implementation of ‘GGIR’ and ‘sleepreg’ to estimate sleep-wake state is described in our previous work [1]. Estimation of daily sleep onset and offset from accelerometer data were made using ‘GGIR’, Version 2.0-0 [2,3], described at: <https://cran.r-project.org/web/packages/GGIR/GGIR.pdf>. Sleep-wake estimation of GGIR has been validated against both sleep diaries polysomnography [3]. Specified GGIR parameters can be found in our ‘sleepreg’ package on Github [<https://github.com/dpwindred/sleepreg>].

Naps and fragmented sleep patterns were accounted for in our sleep-wake state estimation. ‘GGIR’ assumed only one sleep episode per day, and defined wake after sleep onset (WASO) as any epoch within each ‘sleep window’ that did not contain sustained accelerometer inactivity. ‘Naps’ were defined as any interval of 30 minutes or longer with at least 95% sustained inactivity outside GGIR-defined sleep windows. WASO was defined as any interval of 30 minutes or longer without sustained inactivity, inside GGIR-defined sleep windows. Sustained inactivity was defined by GGIR as any continuous period of 5 minutes or longer where the angle of the device relative to the z-axis did not change by more than 5 degrees.

## **S1.7. Sleep Regularity Index (SRI) calculation**

SRI scores were calculated with the following:

$$SRI=-100+200\left( 1-\frac{1}{N_{v}}\sum_{i=1}^{N} \left| s_{i}-s_{i+C} \right| \right)$$

Sleep-wake state was represented by $s_{i}=1$ for wake, $s_{i}=0$ for sleep. Excluded epochs were represented by $s_{i}=NA$. Number of valid epoch-by-epoch comparisons were represented by $N_{v}$, defined as comparisons where $s_{i}\neq NA$ and $s_{i+c}\neq NA$. For any epoch $i$ where $s_{i}=NA$ or $s_{i+c}= NA$, $\left| s_{i}-s_{i+c} \right|=0$. Subscript $i$ represented each epoch from recording start to 24h prior to recording end, such that at:

$$i=1, t_{1}=0$$

$$i=2, t_{2}=E$$

$$i=3, t_{3}=2E$$

_⁞_

$$i=C, t_{C}=E\left( C-1 \right)=24$$

_⁞_

$$i=N, t_{N}=E(N-1)=t_{max}-24$$

Time (h) was represented by $t_{i}$ , epoch length was represented by $E$, total recording length was represented by $t_{max}$ , and number of epochs within one 24 h interval was represented by $C$.The possible range of SRI scores is -100 to 100, where zero represents random sleep-wake patterns. Scores typically do not fall below zero.

## **S1.8. Cleaning and exclusions**

Of an initial sample of 103,104 participants, 98,027 had at least one day of valid sleep-wake data, as defined by GGIR (median [IQR] of 6.95 [6.78-6.99] days of data across participants). Invalid days were defined by GGIR as any interval from noon-noon with >4-h of data classified as missing, clipped, or non-wear. In the remaining sample of 98,027 participants there were no miscalculated nights in 65,627, one night in 27,004, two nights in 4,300, three nights in 764, four nights in 235, five nights in 79, and six nights in 18. Miscalculated nights were defined in our previous work , and were based on sustained inactivity within 1.5 h intervals both before and after onset and offset of GGIR’s estimated daily sleep windows. Miscalculated nights were excluded on a 12-noon to 12-noon basis. Participants had a median (IQR) of 6.92 (5.90 - 6.99) nights of data remaining after exclusion of miscalculated nights. SRI scores required at least 120 h (five days) of 24 h-separated epoch pairs for calculation. This criterion was met in 60,977 participants, who formed the final sample for analysis. Outlying high (>97) and low (<20) SRI scores were visually inspected. Sleep duration was extracted in this sample of 60,977 participants, as the average duration across remaining study days after exclusion of miscalculated nights.

# **S2. Supplementary Results**


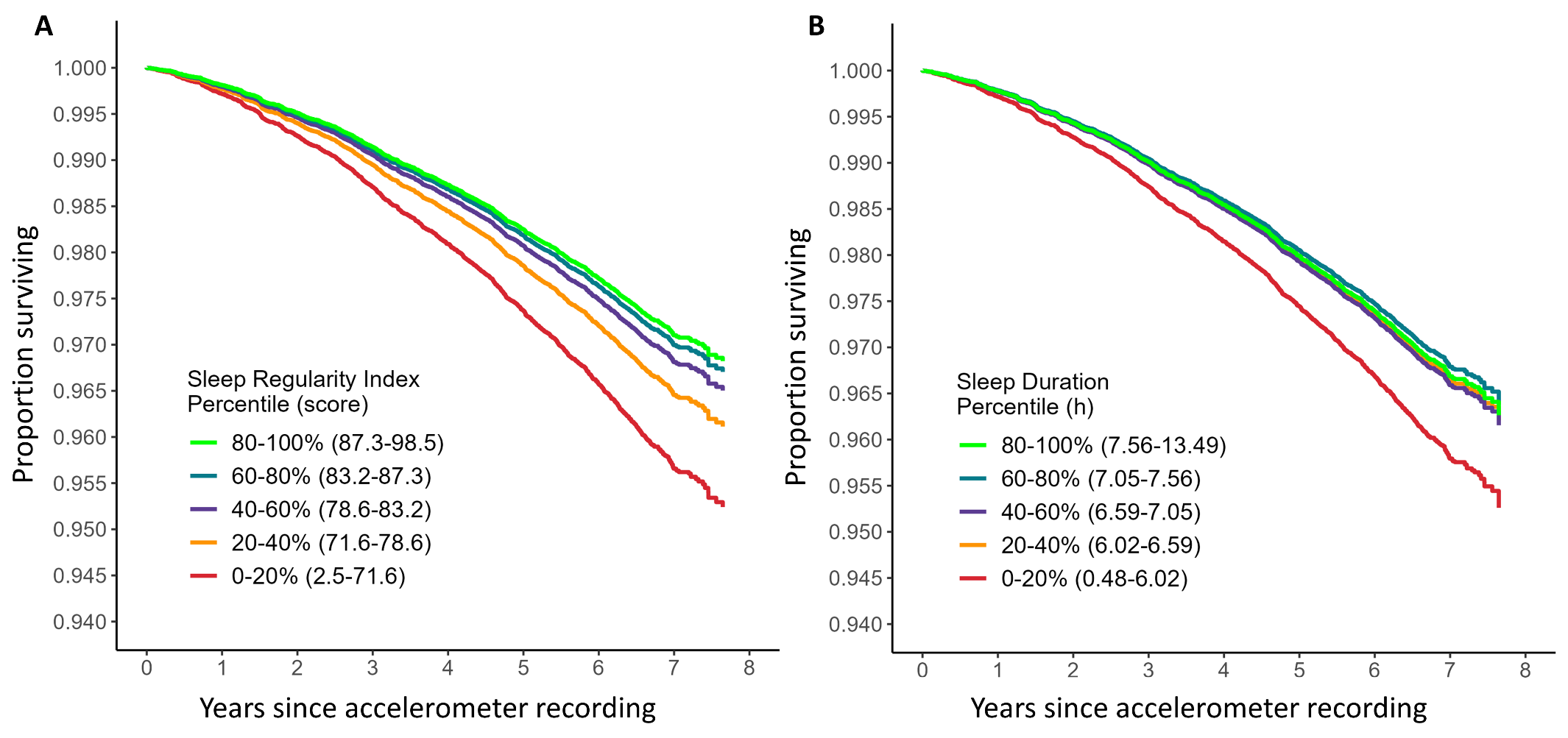


## **Figure S2.1.** Cumulative survival of participants (fully-adjusted)

## **Results S2.2. Comparison of equivalent sleep regularity and sleep duration models**

The probability of minimizing information loss between Models 1 and 2 (see Table S1.5.) was calculated by comparing Akaike Information Criteria, using the following:

$$P=e^{\left( \frac{{AIC}_{SRI}-{AIC}_{duration}}{2} \right)}$$

$P$ represented the probability that the sleep duration model (Model 2) minimized information loss compared to the corresponding sleep regularity model (Model 1). For the minimally adjusted model, ${AIC}_{SRI}$ = 38950.13, ${AIC}_{duration}$ = 39034.94, and $P$ = 3.84 x 10^-19^. For the fully adjusted model, ${AIC}_{SRI}$ = 29009.47, ${AIC}_{duration}$ = 29019.98, and $P$ = 0.005.

| **Table S2.3.** Association between SRI and sleep duration | | | | | | | |
| --- | --- | --- | --- | --- | --- | --- | --- |
| **Model** | **Parameter** | **Estimate** | **Std. Error** | **p** | **Adj. R^2^** | **Model Syntax** | |
| Linear | Intercept | 57.72 | 0.28 | <.001 | 0.085 | SRI ~ sleep_duration | |
|  | p1 | 3.111 | 0.04 | <.001 |  |  | |
| Quadratic | Intercept | 78.78 | 0.04 | <.001 | 0.118 | SRI ~ poly(sleep_duration,2) | |
|  | p1 | 771.5 | 10.0 | <.001 |  |  | |
|  | p2 | -479.1 | 10.0 | <.001 |  |  | |
| Cubic | Intercept | 78.78 | 0.04 | <.001 | 0.124 | SRI ~ poly(sleep_duration,3) | |
|  | p1 | 771.5 | 10.0 | <.001 |  | |  |
|  | p2 | -479.1 | 10.0 | <.001 |  | |  |
|  | p3 | -206.5 | 10.0 | <.001 |  | |  |
| Parameters *p1*, *p2*, and *p3* are coefficients for linear, quadratic, and cubic terms. All models were implemented using the *lm()* function for linear least-squares regression. | | | | | | | |

**Table S2.4.** Hazard of mortality after adjustment for midsleep timing

|  |  | HR [95% CI] |  |
| --- | --- | --- | --- |
|  | Percentile | Fully adjusted | Fully adjusted + Midsleep |
| Model 1: SRI | SRI 0-20% (ref.) | - | - |
|  | SRI 20-40% | 0.80 [0.69-0.93]** | 0.79[0.68-0.92]** |
|  | SRI 40-60% | 0.75 [0.64-0.88]*** | 0.74[0.63-0.86]*** |
|  | SRI 60-80% | 0.72 [0.61-0.84]*** | 0.70[0.60-0.83]*** |
|  | SRI 80-100% | 0.70 [0.59-0.83]*** | 0.68[0.58-0.81]*** |
|  | Midsleep 0-20% | - | 0.83[0.70-0.99]* |
|  | Midsleep 20-40% | - | 0.87[0.74-1.03] |
|  | Midsleep 40-60% | - | 0.96[0.82-1.12] |
|  | Midsleep 60-80% | - | 0.96[0.82-1.11] |
|  | Midsleep 80-100% (ref.) | - | - |
| Model 2: Duration | Duration 0-20% (ref.) | - | - |
|  | Duration 20-40% | 0.82 [0.70-0.97]* | 0.82[0.70-0.97]* |
|  | Duration 40-60% | 0.83 [0.71-0.97]* | 0.83[0.71-0.97]* |
|  | Duration 60-80% | 0.76 [0.65-0.90]** | 0.76[0.65-0.90]*** |
|  | Duration 80-100% | 0.76 [0.65-0.89]*** | 0.76[0.65-0.90]*** |
|  | Midsleep 0-20% | - | 0.87[0.73-1.04] |
|  | Midsleep 20-40% | - | 0.89[0.76-1.05] |
|  | Midsleep 40-60% | - | 0.98[0.84-1.14] |
|  | Midsleep 60-80% | - | 0.97[0.83-1.12] |
|  | Midsleep 80-100% (ref.) | - | - |
| Model 3: SRI + Duration | SRI 0-20% (ref.) | - | - |
|  | SRI 20-40% | 0.83 [0.71-0.96]* | 0.82[0.70-0.95]** |
|  | SRI 40-60% | 0.78 [0.66-0.92]** | 0.77[0.65-0.90]** |
|  | SRI 60-80% | 0.75 [0.64-0.89]** | 0.74[0.62-0.88]*** |
|  | SRI 80-100% | 0.74 [0.62-0.89]*** | 0.73[0.61-0.87]*** |
|  | Duration 0-20% (ref.) | - | - |
|  | Duration 20-40% | 0.88 [0.75-1.04] | 0.89[0.75-1.04] |
|  | Duration 40-60% | 0.90 [0.77-1.07] | 0.91[0.77-1.07] |
|  | Duration 60-80% | 0.84 [0.71-0.99]* | 0.84[0.71-1.00]* |
|  | Duration 80-100% | 0.84 [0.71-0.99]* | 0.85[0.72-1.00]* |
|  | Midsleep 0-20% | - | 0.84[0.70-1.00]* |
|  | Midsleep 20-40% | - | 0.88[0.75-1.04] |
|  | Midsleep 40-60% | - | 0.97[0.83-1.14] |
|  | Midsleep 60-80% | - | 0.97[0.84-1.13] |
|  | Midsleep 80-100% (ref.) | - | - |
| Fully adjusted + Midsleep model comparisons: AIC Model 1 = 29011.3, AIC Model 2 = 29024.4, P = 0.001; Likelihood ratio test Model 1 vs. Model 3: Χ^2^(4) = 5.50, p =.24.  Midsleep was split into quintiles representing early (0-20%) through to late (80-100%) sleep timing. Each quintile was defined with reference to the sample average midsleep time of 03:49, as follows: -11.7 to -0.79 h (0-20%); -0.79 to -0.19 h (20-40%); -0.19 to 0.30 h (40-60%); 0.30 to 0.82 h (60-80%); and 0.82 to 11.8 h (80-100%). The group with the latest sleep timing was defined as the referent group.  Example model syntax for the ‘Fully adjusted + Midsleep’ column is as follows: *Surv(time, death) ~ SRI + age + sex + ethnicity + physical_activity + employment + income + deprivation + social_activity + social_visits + smoking + urbanicity + shiftwork + medication + midsleep.* | | | |

|  |  |  | |  | HR [95% CI] | |  | |  | |
| --- | --- | --- | --- | --- | --- | --- | --- | --- | --- | --- |
|  | Percentile | Diabetes | Cancer | | | Vascular | | BMI | | Cholesterol Ratio |
| Model 1: SRI | SRI 0-20% | - | - | | | - | | - | | - |
|  | SRI 20-40% | 0.81[0.70-0.94]** | 0.79[0.68-0.92]** | | | 0.80[0.69-0.93]** | | 0.81[0.70-0.95]** | | 0.81[0.69-0.96]* |
|  | SRI 40-60% | 0.76[0.65-0.89]*** | 0.74[0.64-0.87]*** | | | 0.75[0.64-0.87]*** | | 0.76[0.65-0.89]*** | | 0.75[0.63-0.89]*** |
|  | SRI 60-80% | 0.73[0.62-0.86]*** | 0.71[0.61-0.84]*** | | | 0.72[0.61-0.85]*** | | 0.73[0.62-0.87]*** | | 0.72[0.60-0.86]*** |
|  | SRI 80-100% | 0.71[0.60-0.84]*** | 0.70[0.59-0.83]*** | | | 0.70[0.59-0.82]*** | | 0.72[0.61-0.85]*** | | 0.71[0.60-0.85]*** |
| Model 2: Duration | Duration 0-20% | - | - | | | - | | - | | - |
|  | Duration 20-40% | 0.83[0.71-0.98]* | 0.82[0.70-0.97]* | | | 0.83[0.70-0.97]* | | 0.83[0.71-0.98]* | | 0.80[0.67-0.94]** |
|  | Duration 40-60% | 0.84[0.72-0.99]* | 0.83[0.71-0.97]* | | | 0.83[0.71-0.97]* | | 0.85[0.72-0.99]* | | 0.83[0.70-0.99]* |
|  | Duration 60-80% | 0.78[0.66-0.91]** | 0.77[0.65-0.90]** | | | 0.77[0.65-0.90]** | | 0.78[0.66-0.92]** | | 0.73[0.61-0.86]*** |
|  | Duration 80-100% | 0.77[0.66-0.90]** | 0.77[0.65-0.90]*** | | | 0.76[0.65-0.89]*** | | 0.79[0.67-0.92]** | | 0.72[0.61-0.86]*** |
| Model 3: SRI + Duration | SRI 0-20% | - | - | | | - | | - | | - |
|  | SRI 20-40% | 0.83[0.71-0.97]* | 0.81[0.70-0.95]** | | | 0.83[0.71-0.96]* | | 0.84[0.72-0.97]* | | 0.84[0.72-0.99]* |
|  | SRI 40-60% | 0.79[0.67-0.93]** | 0.78[0.66-0.91]** | | | 0.78[0.66-0.92]** | | 0.79[0.67-0.93]** | | 0.79[0.66-0.94]** |
|  | SRI 60-80% | 0.76[0.64-0.90]** | 0.75[0.63-0.89]*** | | | 0.75[0.64-0.89]** | | 0.77[0.65-0.91]** | | 0.76[0.64-0.92]** |
|  | SRI 80-100% | 0.75[0.63-0.89]** | 0.75[0.63-0.89]** | | | 0.74[0.62-0.88]*** | | 0.76[0.64-0.91]** | | 0.77[0.64-0.93]** |
|  | Duration 0-20% | - | - | | | - | | - | | - |
|  | Duration 20-40% | 0.89[0.76-1.05] | 0.88[0.75-1.04] | | | 0.88[0.75-1.04] | | 0.89[0.75-1.04] | | 0.85[0.71-1.01] |
|  | Duration 40-60% | 0.92[0.78-1.08] | 0.90[0.77-1.07] | | | 0.90[0.77-1.06] | | 0.91[0.78-1.08] | | 0.90[0.76-1.07] |
|  | Duration 60-80% | 0.85[0.72-1.01] | 0.85[0.71-1.00]* | | | 0.84[0.71-1.00]* | | 0.85[0.72-1.01] | | 0.79[0.66-0.95]* |
|  | Duration 80-100% | 0.84[0.72-1.00]* | 0.84[0.71-0.99]* | | | 0.83[0.71-0.98]* | | 0.86[0.73-1.01] | | 0.79[0.66-0.94]** |
| Each column contains output from three individual models. Each model was implemented as the fully-adjusted model described in the main text, plus the individual covariate specified by the column header (e.g., for Model 1:SRI, and the Diabetes column, the following model syntax was implemented: *Surv(time, death) ~ SRI + age + sex + ethnicity + physical_activity + employment + income + deprivation + social_activity + social_visits + smoking + urbanicity + shiftwork + medication + diabetes*). | | | | | | | | | | |

## **Table S2.5.** Hazard of mortality after adjustment for baseline physical health

## **Table S2.6.** Hazard of mortality after adjustment for baseline mental health

|  |  | HR [95% CI] | | | | | |
| --- | --- | --- | --- | --- | --- | --- | --- |
|  | Percentile | Depressed mood | Unenthusiasm / disinterest | Tenseness / restlessness | Tiredness / lethargy | Visited GP for mental health | Visited psychiatrist for mental health |
| Model 1: SRI | SRI 0-20% | - | - | - | - | - | - |
|  | SRI 20-40% | 0.80[0.69-0.93]** | 0.79[0.68-0.92]** | 0.81[0.70-0.94]** | 0.81[0.70-0.94]** | 0.81[0.69-0.93]** | 0.81[0.70-0.94]** |
|  | SRI 40-60% | 0.74[0.63-0.87]*** | 0.75[0.64-0.88]*** | 0.74[0.63-0.87]*** | 0.74[0.63-0.87]*** | 0.75[0.64-0.88]*** | 0.76[0.65-0.89]*** |
|  | SRI 60-80% | 0.71[0.60-0.84]*** | 0.71[0.60-0.84]*** | 0.70[0.59-0.83]*** | 0.71[0.60-0.84]*** | 0.72[0.61-0.84]*** | 0.73[0.62-0.86]*** |
|  | SRI 80-100% | 0.70[0.59-0.83]*** | 0.70[0.59-0.83]*** | 0.69[0.59-0.82]*** | 0.70[0.59-0.83]*** | 0.70[0.59-0.83]*** | 0.71[0.60-0.84]*** |
| Model 2: Duration | Duration 0-20% | - | - | - | - | - | - |
|  | Duration 20-40% | 0.81[0.69-0.95]** | 0.83[0.71-0.98]* | 0.81[0.69-0.95]** | 0.81[0.69-0.95]* | 0.82[0.70-0.96]* | 0.84[0.71-0.98]* |
|  | Duration 40-60% | 0.82[0.70-0.97]* | 0.82[0.70-0.97]* | 0.82[0.70-0.97]* | 0.84[0.71-0.98]* | 0.83[0.71-0.98]* | 0.84[0.72-0.99]* |
|  | Duration 60-80% | 0.77[0.66-0.91]** | 0.77[0.65-0.90]** | 0.77[0.65-0.90]** | 0.77[0.65-0.90]** | 0.77[0.65-0.90]** | 0.77[0.66-0.91]** |
|  | Duration 80-100% | 0.77[0.65-0.90]** | 0.75[0.64-0.88]*** | 0.75[0.64-0.88]*** | 0.76[0.65-0.89]*** | 0.76[0.65-0.89]*** | 0.77[0.66-0.90]** |
| Model 3: SRI + | SRI 0-20% | - | - | - | - | - | - |
| Duration | SRI 20-40% | 0.82[0.71-0.96]* | 0.81[0.70-0.95]** | 0.84[0.72-0.97]* | 0.83[0.71-0.97]* | 0.83[0.71-0.97]* | 0.83[0.71-0.97]* |
|  | SRI 40-60% | 0.77[0.66-0.91]** | 0.78[0.66-0.92]** | 0.78[0.66-0.92]** | 0.77[0.65-0.91]** | 0.78[0.67-0.92]** | 0.79[0.67-0.93]** |
|  | SRI 60-80% | 0.75[0.63-0.89]** | 0.75[0.63-0.89]** | 0.74[0.62-0.88]*** | 0.75[0.63-0.89]** | 0.75[0.64-0.89]** | 0.76[0.64-0.90]** |
|  | SRI 80-100% | 0.75[0.62-0.89]** | 0.74[0.62-0.89]** | 0.74[0.62-0.88]*** | 0.74[0.62-0.89]** | 0.74[0.62-0.89]*** | 0.75[0.63-0.90]** |
|  | Duration 0-20% | - | - | - | - | - | - |
|  | Duration 20-40% | 0.86[0.73-1.02] | 0.89[0.76-1.05] | 0.87[0.74-1.02] | 0.87[0.74-1.02] | 0.88[0.74-1.03] | 0.89[0.76-1.05] |
|  | Duration 40-60% | 0.90[0.76-1.06] | 0.90[0.76-1.06] | 0.90[0.76-1.06] | 0.91[0.77-1.08] | 0.91[0.77-1.07] | 0.91[0.78-1.08] |
|  | Duration 60-80% | 0.85[0.72-1.01] | 0.85[0.71-1.00] | 0.85[0.71-1.00] | 0.85[0.72-1.00] | 0.84[0.71-1.00]* | 0.85[0.72-1.00] |
|  | Duration 80-100% | 0.84[0.71-1.00]* | 0.83[0.70-0.98]* | 0.83[0.70-0.98]* | 0.84[0.71-0.99]* | 0.83[0.71-0.98]* | 0.84[0.72-0.99]* |
| Each column contains output from three individual models. Each model was implemented as the fully-adjusted model described in the main text, plus the individual covariate specified by the column header (e.g., for Model 1:SRI, and the Depressed mood column, the following model syntax was implemented: *Surv(time, death) ~ SRI + age + sex + ethnicity + physical_activity + employment + income + deprivation + social_activity + social_visits + smoking + urbanicity + shiftwork + medication + depressed_mood*). | | | | | | | |

## **Table S2.7.** Intra-individual variability in sleep onset and offset timing according to SRI percentile

| SRI score | SRI percentile | Sleep onset IIV | | Sleep offset IIV | |
| --- | --- | --- | --- | --- | --- |
|  |  | Mean ± SD | Median (IQR) | Mean ± SD | Median (IQR) |
| 2.5-71.6 | 0-20% | 1.83±1.16 | 1.58 (1.05-2.25) | 1.75±1.26 | 1.43 (0.97-2.05) |
| 71.6-78.6 | 20-40% | 1.22±0.71 | 1.08 (0.75-1.51) | 1.20±0.74 | 1.06 (0.76-1.45) |
| 78.6-83.2 | 40-60% | 0.96±0.56 | 0.85 (0.61-1.15) | 1.00±0.55 | 0.91 (0.66-1.21) |
| 83.2-87.3 | 60-80% | 0.77±0.47 | 0.68 (0.49-0.92) | 0.87±0.48 | 0.79 (0.59-1.04) |
| 87.3-98.5 | 80-100% | 0.55±0.38 | 0.47 (0.33-0.66) | 0.68±0.41 | 0.61 (0.45-0.82) |
| Intra-individual variability (IIV) was calculated as the standard deviation of sleep onset/offset times within each participant’s one-week recording, using the same set of valid study days that were also used to calculate SRI and sleep duration. | | | | | |

# **S3. STROBE Checklist**

STROBE Statement—Checklist of items that should be included in reports of ***cohort studies***

|  | Item No | Recommendation | Manuscript Section |
| --- | --- | --- | --- |
| **Title and abstract** | 1 | (*a*) Indicate the study’s design with a commonly used term in the title or the abstract | - Title |
|  |  | (*b*) Provide in the abstract an informative and balanced summary of what was done and what was found | - Abstract |
| Introduction | | |  |
| Background/rationale | 2 | Explain the scientific background and rationale for the investigation being reported | - Introduction, Paragraphs 1-2 |
| Objectives | 3 | State specific objectives, including any prespecified hypotheses | - Introduction, Paragraph 3 |
| Methods | | |  |
| Study design | 4 | Present key elements of study design early in the paper | - Introduction, Paragraph 3 - Methods: Overview |
| Setting | 5 | Describe the setting, locations, and relevant dates, including periods of recruitment, exposure, follow-up, and data collection | - Abstract - Methods: Overview - Results: Participant characteristics - Supporting Information S1.1. |
| Participants | 6 | (*a*) Give the eligibility criteria, and the sources and methods of selection of participants. Describe methods of follow-up | - Methods: Overview - Supporting Information S1.1., S1.2. |
|  |  | (*b*) For matched studies, give matching criteria and number of exposed and unexposed | NA |
| Variables | 7 | Clearly define all outcomes, exposures, predictors, potential confounders, and effect modifiers. Give diagnostic criteria, if applicable | - Methods: Sleep regularity and sleep duration - Methods: Mortality records - Methods: Covariates - Supporting Information S1.2., 1.3., 1.6., 1.7. |
| Data sources/ measurement | 8* | For each variable of interest, give sources of data and details of methods of assessment (measurement). Describe comparability of assessment methods if there is more than one group | - Methods: Covariates - Supporting Information S1.2., 1.3. |
| Bias | 9 | Describe any efforts to address potential sources of bias | - Methods: Sleep regularity and sleep duration - Methods: Statistical analyses - Supporting Information S1.6,1.8 |
| Study size | 10 | Explain how the study size was arrived at | NA |
| Quantitative variables | 11 | Explain how quantitative variables were handled in the analyses. If applicable, describe which groupings were chosen and why | - Methods: Statistical analyses - Supporting information S1.3. |
| Statistical methods | 12 | (*a*) Describe all statistical methods, including those used to control for confounding | - Methods: Statistical analyses - Supporting information S1.4,.1.5., 2.2., 2.3. |
|  |  | (*b*) Describe any methods used to examine subgroups and interactions | - Methods: Statistical analyses - Supporting information S2.4-6. |
|  |  | (*c*) Explain how missing data were addressed | - Supplementary S1.6., 1.8. |
|  |  | (*d*) If applicable, explain how loss to follow-up was addressed | NA |
|  |  | (*e*) Describe any sensitivity analyses | NA |
| Results | | |  |
| Participants | 13* | (a) Report numbers of individuals at each stage of study—eg numbers potentially eligible, examined for eligibility, confirmed eligible, included in the study, completing follow-up, and analysed | - Methods: Overview - Supporting Information S1.8. |
|  |  | (b) Give reasons for non-participation at each stage | - Methods: Overview |
|  |  | (c) Consider use of a flow diagram | NA |
| Descriptive data | 14* | (a) Give characteristics of study participants (eg demographic, clinical, social) and information on exposures and potential confounders | - Results: Participant characteristics - Results: Table 1 |
|  |  | (b) Indicate number of participants with missing data for each variable of interest | - Supporting information: S1.8. |
|  |  | (c) Summarise follow-up time (eg, average and total amount) | - Abstract - Results: Participant characteristics |
| Outcome data | 15* | Report numbers of outcome events or summary measures over time | - Results: Figure 1 |
| Main results | 16 | (*a*) Give unadjusted estimates and, if applicable, confounder-adjusted estimates and their precision (eg, 95% confidence interval). Make clear which confounders were adjusted for and why they were included | - Results: Tables 1-3 - Supporting Information: S2.4-6 |
|  |  | (*b*) Report category boundaries when continuous variables were categorized | - Methods: Statistical analyses |
|  |  | (*c*) If relevant, consider translating estimates of relative risk into absolute risk for a meaningful time period | NA |
| Other analyses | 17 | Report other analyses done—eg analyses of subgroups and interactions, and sensitivity analyses | - Methods: Statistical analyses - Supporting Information: S2.4-6 |
| Discussion | | |  |
| Key results | 18 | Summarise key results with reference to study objectives | - Discussion: Paragraph 1 |
| Limitations | 19 | Discuss limitations of the study, taking into account sources of potential bias or imprecision. Discuss both direction and magnitude of any potential bias | - Discussion: Paragraph 7 |
| Interpretation | 20 | Give a cautious overall interpretation of results considering objectives, limitations, multiplicity of analyses, results from similar studies, and other relevant evidence | - Discussion: Paragraphs 2-6, 8 |
| Generalisability | 21 | Discuss the generalisability (external validity) of the study results | - Discussion: Paragraph 7 |
| Other information | | |  |
| Funding | 22 | Give the source of funding and the role of the funders for the present study and, if applicable, for the original study on which the present article is based | NA |

# **References**

1. Windred DP, Jones SE, Russell A, et al. Objective assessment of sleep regularity in 60 000 UK Biobank participants using an open-source package. Sleep. 2021; 44 (12): zsab254.

2. Migueles JH, Rowlands AV, Huber F, Sabia S, van Hees VT. GGIR: a research community–driven open source R package for generating physical activity and sleep outcomes from multi-day raw accelerometer data. JMPB. 2019; 2 (3): 188-196.

3. van Hees VT, Sabia S, Jones SE, et al. Estimating sleep parameters using an accelerometer without sleep diary. Sci Rep. 2018; 8 (1): 1-11.
